# The role of stroke emergency specialist nurses in revascularization therapy for acute ischemic stroke

**DOI:** 10.1097/MD.0000000000046438

**Published:** 2025-12-26

**Authors:** Xiaoting Chen, Feng Wang

**Affiliations:** a Department of Emergency, Cangzhou Integrated Traditional Chinese and Western Medicine Hospital, Cangzhou, Hebei Province, China; b Department of Joint Trauma, Cangzhou Integrated Traditional Chinese and Western Medicine Hospital, Cangzhou, Hebei Province, China.

**Keywords:** acute ischemic stroke revascularization treatment, neurological function, stroke emergency nurses

## Abstract

This study aims to examine the impact of stroke emergency nurses’ involvement in the treatment of acute ischemic stroke through revascularization. This retrospective study analyzed 280 patients with acute ischemic stroke who were admitted to the emergency department of Cangzhou Integrated Traditional Chinese and Western Medicine Hospital between January 2021 and June 2023. According to the type of emergency management received, patients were divided into 2 groups: 140 patients who received standard emergency care (control group) and 140 patients whose treatment process additionally involved stroke emergency specialist nurses (intervention group). Clinical data, including demographic characteristics, revascularization time intervals, neurological function after revascularization, and overall therapeutic outcomes, were collected from the hospital’s medical records and compared between the 2 groups. The intervention group exhibited shorter time intervals for each treatment stage compared to the control group (*P* < .001). After 7 and 30 days of revascularization, the National Institute of Health Stroke Scale scores of the intervention group were lower than those of the control group (*P* < .05). Additionally, after 90 days, the modified Rankin scale scores of the intervention group were also significantly lower (*P* < .001), indicating better recovery. Involving stroke emergency nurses in the revascularization process for acute ischemic stroke patients leads to reduced treatment time at various stages and improved patient outcomes.

## 1. Introduction

Acute ischemic stroke constitutes the most common type of stroke. It bears high morbidity, mortality, and disability.^[1]^ About >75% of patients with stroke have different degrees of low nutritional level and motor dysfunction in their prognosis. It even affects normal life walking and secondary related complications.^[2]^ Some relevant literature shows that the efficacy of revascularization therapy is highly time-dependent. The shorter the time from onset to revascularization, the better the clinical prognosis.^[3,4]^ Therefore, how to conduct rapid and active revascularization of patients at the early stage of onset as well as save the ischemic semi-dark band within the time window has become the main principle. The impact of reducing the area of cerebral infarction and improving the rate of vascularization is of great significance for research and therapeutic value.^[5]^

In cerebrovascular recanalization therapy, nurses are prominent members of the clinical team. They bear important responsibilities. Those nurses need to conduct a comprehensive assessment of patients in the acute phase. They also need to identify and deal with complications in a timely manner. In addition, they assist doctors in cerebrovascular recanalization therapy operations, monitor the patient’s condition changes, and provide necessary nursing support. Studies have shown that configuring stroke emergency nurses to participate in the entire process of patients’ intravenous thrombolysis treatment in the early stage facilitates continuous quality improvement.^[6]^ In previous studies, different nursing models have been used as an entry point to explore the impact on stroke-related complications and sequelae.^[7–9]^ Only a few studies have examined the role of stroke emergency nurses in participating in acute ischemic stroke treatment to evaluate and analyze the effects of revascularization therapy.

This study takes acute ischemic stroke patients’ revascularization therapy as an entry point. It studies and analyzes the clinical effects of stroke emergency nurses’ participation in revascularization therapy. It also discusses the effects on patients’ recovery and disease prognosis. Furthermore, it provides reference value for future improvement of clinical treatment protocols for acute ischemic stroke, and further standardization of specialized nursing training/education.

## 2. Materials and methods

### 2.1. General information and grouping

This retrospective study was approved by the Ethics Committee of Cangzhou Integrated Traditional Chinese and Western Medicine Hospital (Approval No. CZX2024031). From January 2021 to June 2023, a total of 280 patients diagnosed with acute ischemic stroke and treated with revascularization therapy in the hospital’s emergency department were included. As this study involved a retrospective review of anonymized medical records, the requirement for written informed consent was waived by the Ethics Committee in accordance with the Declaration of Helsinki and institutional guidelines. According to the type of emergency management recorded in the medical files, patients were divided into 2 groups: the control group (n = 140), who received standard emergency care, and the intervention group (n = 140), in which stroke emergency specialist nurses participated in the treatment process. Demographic and clinical baseline data, including gender, age, smoking history, hypertension, diabetes mellitus, and hyperlipidemia, were collected from the hospital’s medical records.

Inclusion criteria: Adherence to the 2018 Guidelines for the Early Management of Acute Ischemic Stroke,^[10]^ with diagnoses confirmed by cranial imaging and relevant clinical signs and symptoms. Age over 18 years. Patients who underwent endovascular mechanical thrombolysis. Successful revascularization confirmed by the modified cerebral infarction thrombolysis score. No intracranial hemorrhage detected on computed tomography (CT) scans within 24 hours after the procedure.

Exclusion criteria: Concurrent subarachnoid hemorrhage. Major surgery performed within 90 days prior to stroke onset. Incomplete data. Contraindications to surgery.

### 2.2. Treatment

In the control group, patients received standard emergency care. Upon arrival at the emergency department, they were initially assessed by a prescreening nurse and referred to the appropriate clinic for necessary auxiliary examinations. After the results were obtained, the attending physicians conducted a professional evaluation. Patients and their families were then presented with treatment options, and after obtaining informed consent, preoperative preparations for thrombolysis, if required, were initiated.

In the intervention group, in addition to the standard care, a stroke emergency specialist nurse was assigned to assist in the treatment process.

These nurses were registered nurses with >5 years of experience in neurology or emergency medicine who had completed formal stroke emergency certification training organized by the hospital’s stroke center. The training covered acute stroke assessment, National Institute of Health Stroke Scale (NIHSS) scoring, intravenous thrombolysis assistance, neurological monitoring, complication management, and interdepartmental coordination skills. The primary responsibilities of the stroke emergency specialist nurses included: rapid triage and early identification of suspected stroke cases upon patient arrival; coordination among emergency physicians, neurologists, radiology, and laboratory departments to expedite diagnostic and treatment procedures; preparation and administration assistance for thrombolytic agents and related revascularization materials; continuous monitoring of vital signs and neurological function during and after the procedure; and providing patient and family education about stroke management and time sensitivity.

During the intravenous thrombolysis procedure, the workflow was as follows: Following the initial consultation, the nurse triaged the patient and coordinated with a specialist. The physician alerted the radiology department to prepare for examinations, while the nurse established intravenous access for the patient. After the physician made a diagnosis, they informed the patient or family about the condition and assessed their willingness to proceed with thrombolysis. The stroke team was notified, and the nurse prepared the necessary medication. The nurse and physician then accompanied the patient to the CT room. During the transfer, the specialist explained the importance of thrombolysis and reiterated the critical time window to the family. Once informed consent was signed, thrombolysis was administered at the bedside in the CT room. Additional doses were administered after the patient was moved to the resuscitation room. Following the procedure, multimodal imaging was conducted, and the results were communicated to the relevant teams to prepare the arterial and catheterization rooms. The patient was then transferred to the resuscitation room for further monitoring. Both groups of patients were observed for 90 days after treatment.

### 2.3. Indicators for evaluating revascularization elapsed time

The time taken for each stage of the emergency process was recorded and compared between the 2 groups. This included the duration from patient admission to emergency room reception (door to preadmission [DTP]), the time from admission to the issuance of the CT report (door to imaging [DTI]), the time from admission to receiving laboratory diagnostic results (door to laboratory [DTL]), and the time from admission to the initiation of thrombolysis (door to needle [DTN]).

### 2.4. Comparison of nerve function after revascularization

The NIHSS was employed to evaluate the neurological function of patients after revascularization.^[11]^ Assessments were conducted at 24 hours, 7 days, and 30 days post-procedure, with neurological function being recorded at each time point. The ability of the 2 groups to perform daily activities was assessed at 90 days postoperatively using the modified Rankin scale (mRS), a widely used and validated tool for assessing disability in stroke patients.^[12]^ A lower score on both the NIHSS and mRS indicated better neurological function and greater independence.

### 2.5. Indicators for evaluating therapeutic effects after revascularization

The evaluation indexes of treatment effect included the incidence of intracranial hemorrhage (symptomatic intracranial hemorrhage [sICH]) after revascularization, the incidence of cerebral herniation within 90 days, and the mortality rate within 90 days. The incidence of sICH was defined as the conversion of intracranial hemorrhage within 36 to 48 hours after endovascular thrombolysis.^[13]^ Among them, sICH was defined as imaging studies suggesting hemorrhagic transformation within 36 to 48 hours after endovascular thrombectomy.^[13]^

### 2.6. Statistical processing

Data analysis was performed using SPSS 23.0 statistical software (IBM SPSS Statistics, IBM Corp., Armonk ). Categorical data were presented as percentages, and the χ² test was applied. For measurement data conforming to a normal distribution, results were expressed as “$\bar{x}\pm s$” and analyzed using the *t* test. Non-normally distributed data or those with unequal variances were reported as medians. A *P* value of < .05 was considered statistically significant.

## 3. Result

### 3.1. Comparison of baseline data between the 2 groups of patients

Among the 280 patients who underwent revascularization therapy, the control group consisted of 67 males and 73 females with an average age of 66.19 ± 5.41 years. In this group, 50 patients had a history of smoking, 48 had hypertension, 20 had diabetes mellitus, and 66 had hyperlipidemia. The intervention group comprised 72 males and 68 females, with an average age of 66.34 ± 6.47 years. In this group, 48 patients had a history of smoking, 38 had hypertension, 24 had diabetes mellitus, and 60 had hyperlipidemia. There were no statistically significant differences in baseline characteristics between the 2 groups (*P* > .05; see Table 1).

**Table 1:** Comparison of baseline data of patients with 2 groups.

Characteristics	Control group N = 140	Intervention group N = 140	Statistic	*P* value
Age (yr), $\bar{x}\pm s$	66.19 ± 5.41	66.34 ± 6.47	−0.350	.727
Male, n (%)	67 (47.36)	72 (51.43)	0.357	.550
Stroke risk factor, n (%)				
Smoking	50 (35.71)	48 (34.29)	0.063	.802
Hypertension	48 (34.29)	38 (27.14)	1.678	.195
Diabetes mellitus	20 (14.29)	24 (17.14)	0.431	.511
Hyperlipidemia	66 (47.14)	60 (42.86)	0.211	.645

### 3.2. Comparison of time consuming revascularization in 2 groups of patients

As shown in Table 2, the intervention group had shorter times for all stages of the process compared to the control group, including the time from admission to emergency room reception (DTP), time to CT report (DTI), time to laboratory diagnostic report (DTL), and time to thrombolysis initiation (DTN). All time intervals were measured in minutes. The differences between the 2 groups were statistically significant (*P* < .001). Overall, patients in the intervention group spent less time in each consultation and treatment stage than those in the control group.

**Table 2:** Comparison of time consumption of 2 groups.

Index	Control group N = 140	Intervention group N = 140	*t* value	*P* value
Door-to-puncture time	22.11 ± 3.01	11.09 ± 3.78	27.034	<.001
Door-to-imaging time	36.27 ± 5.11	27.51 ± 4.25	22.540	<.001
Door to Laboratory time	70.32 ± 9.12	35.04 ± 7.03	35.179	<.001
Door to needle time	89.79 ± 5.16	37.06 ± 8.32	68.199	<.001

### 3.3. Comparison of neurologic function after revascularization in 2 groups of patients

As indicated in Table 3, after 7 days of revascularization treatment, the NIHSS score of the intervention group was lower (18.93 ± 3.30, *P* < .05). At 30 days, the intervention group’s NIHSS score (12.51 ± 3.25) was significantly lower than that of the control group (17.43 ± 3.43, *P* < .001). Additionally, after 90 days of treatment, the mRS score in the intervention group was 1.29 ± 1.02, reflecting a better prognosis compared to the control group (*P* < .001).

**Table 3:** Comparison of NIHSS and mRS score of patients in 2 groups.

Groups	NIHSS score			90-d mRS
	24 h	7 d	30 d	
Control group N = 140	20.21 ± 3.21	19.13 ± 3.52	17.43 ± 3.43	2.50 ± 1.12
Intervention group N = 140	20.63 ± 3.29	18.93 ± 3.30	12.51 ± 3.25	1.29 ± 1.02
*t* value	−1.267	2.026	12.828	10.705
*P* value	.207	.045	<.001	<.001

mRS = modified Rankin scale, NIHSS = National Institute of Health Stroke Scale.

### 3.4. Comparison of the results of revascularization therapy in 2 groups of patients

As shown in Table 4, following revascularization treatment, the intervention group experienced 3 cases of intracranial hemorrhage, accounting for 2.14% of the total, significantly lower than the 9.29% in the control group. There was 1 case of cerebral hernia in the intervention group (0.71%), which was also significantly lower than the 14.29% observed in the control group. Additionally, over 90 days, there were 11 deaths in the intervention group (7.86%), a notably lower rate compared to 14.29% in the control group (*P* < .001).

**Table 4:** Comparison of treatment results of patients in 2 groups.

Index	Control group N = 140	Experiment group N = 140	*X* ^2^	*P* value
sICH, n (%)	13 (9.29)	3 (2.14)	29.949	<.001
Cerebral hernia (%)	20 (14.29)	1 (0.71)	6.043	.014
Death, n (%)	20 (14.29)	11 (7.86)	71.628	<.001

sICH = symptomatic intracranial hemorrhage.

## 4. Discussion

### 4.1. Acute ischemic stroke care involving stroke emergency nurses reduces work time at all stages of the process

Emergency nurses, who primarily handle acute and critical patients, face high workloads and intense work demands. This requires not only quick emergency response skills but also extensive clinical experience to rapidly identify patient needs. Given the wide range of illnesses and conditions encountered in the emergency department, nurses must possess both strong clinical expertise and comprehensive medical knowledge to respond efficiently in urgent situations. In this study, the intervention group showed shorter times in all stages, including from admission to emergency department reception (DTP), CT report (DTI), laboratory diagnostic report (DTL), and thrombolysis (DTN) compared to the control group (*P* < .001). This indicates that stroke emergency nurses can significantly reduce the time patients spend in care, leading to quicker examinations and treatments. These findings align with the results of Zhou ^[14]^ and other researchers. The inclusion of stroke emergency nurses strengthens the emergency care team, highlights the value of specialized nursing skills, improves the efficiency of stroke treatment, and reduces the time required at each stage of care. This study supports the clinical benefits of involving stroke emergency nurses in the revascularization treatment of acute ischemic stroke patients.

### 4.2. Revascularization with the participation of stroke nurses can improve the neurological function of patients

This study monitored the time spent in each session and evaluated the NIHSS scores of patients at 24 hours, 7 days, and 30 days post-revascularization. The results showed that at both 7 and 30 days, NIHSS scores in the intervention group were lower than those in the control group, indicating less severe neurological deficits. Patients in the intervention group also demonstrated better motor function, limb coordination, sensory function, and language skills. After 90 days of treatment, mRS scores were also assessed, with the intervention group scoring 1.29 ± 1.02 compared to 2.50 ± 1.12 in the control group (*P* < .001). An mRS score of ≤ 2 indicated a good prognosis, while scores between 3 and 6 were considered poor.^[7]^ These findings suggest that patients treated with the participation of stroke emergency nurses experienced better neurological recovery and outcomes, which aligns with previous studies by Xu et al.^[15]^

In addition to NIHSS and mRS scores, this study also tracked the incidence of intracranial hemorrhage, cerebral hernia, and patient mortality 90 days post-revascularization. The intervention group experienced 3 cases of intracranial hemorrhage, 1 case of cerebral hernia, and 11 deaths, all lower than in the control group (*P* < .05). These results indicate that the involvement of stroke nurses not only improved neurological outcomes but also helped reduce associated risk factors and enhanced the overall success rate of treatment.

### 4.3. Participation of stroke emergency nurses in revascularization in acute ischemic stroke has important clinical implications

In summary, stroke emergency specialist nurses play a crucial role in acute ischemic stroke management by shortening the time spent at each treatment stage, reducing neurological deficits, improving recovery outcomes, and lowering the incidence of post-revascularization complications. Compared with similar studies conducted domestically and internationally, our findings are consistent with those of Middleton et al ^[9]^ and Xu et al ^[15]^, which demonstrated that nurse-led or nurse-coordinated stroke emergency models significantly improved treatment efficiency and patient prognosis. However, unlike studies from high-resource centers abroad where stroke nurse systems are well-established, our results highlight the effectiveness of such a model in a regional hospital setting in China, suggesting its feasibility in developing or resource-limited environments.

Nevertheless, the widespread implementation of this model may face several challenges, including the shortage of trained stroke nurses, the need for standardized and sustainable training systems, and increased financial and human resource demands associated with continuous education and team coordination. Addressing these issues will be crucial for promoting this model on a broader scale.

This study has several limitations. First, it was a single-center retrospective observational study, which may limit the generalizability of the findings. Second, the patient population was drawn from one region, potentially introducing regional bias. Third, long-term outcomes beyond 90 days were not assessed, and future studies should include extended follow-up to evaluate functional recovery and quality of life. Moreover, multicenter prospective studies with larger sample sizes and cost-effectiveness analyses are needed to further validate the clinical and economic value of stroke emergency nurse participation in revascularization therapy.

## Acknowledgments

The authors thank all the staff members of Cangzhou Integrated Traditional Chinese and Western Medicine Hospital for their support during the study.

## Author contributions

**Conceptualization:** Xiaoting Chen, Feng Wang.

**Data curation:** Xiaoting Chen, Feng Wang.

**Formal analysis:** Xiaoting Chen, Feng Wang.

**Funding acquisition:** Xiaoting Chen, Feng Wang.

**Investigation:** Xiaoting Chen, Feng Wang.

**Writing – original draft:** Xiaoting Chen, Feng Wang.

**Writing – review & editing:** Xiaoting Chen, Feng Wang.
